# Angiogenesis-Related Markers and Prognosis After Cytoreductive Surgery and Hyperthermic Intraperitoneal Chemotherapy for Metastatic Colorectal Cancer

**DOI:** 10.1245/s10434-015-5023-0

**Published:** 2016-01-04

**Authors:** E. M. V. de Cuba, I. H. J. T. de Hingh, N. R. Sluiter, R. Kwakman, V. M. H. Coupé, J. A. M. Beliën, V. J. Verwaal, W. J. H. J. Meijerink, P. M. Delis-van Diemen, H. J. Bonjer, G. A. Meijer, E. A. te Velde

**Affiliations:** Department of Surgical Oncology, VU University Medical Center Amsterdam, Amsterdam, The Netherlands; Department of Pathology, VU University Medical Center, Amsterdam, The Netherlands; Department of Surgery, Catharina Ziekenhuis Eindhoven, Eindhoven, The Netherlands; Department of Epidemiology and Biostatistics, VU University Medical Center, Amsterdam, The Netherlands; Department of Pathology, Netherlands Cancer Institute, Amsterdam, The Netherlands; Department of General Surgery, Section of Surgical Oncology and Digestive Surgery, VU University Medical Center, Amsterdam, The Netherlands

## Abstract

**Background:**

Patients presenting with peritoneal metastases (PM) of colorectal cancer (CRC) can be curatively treated with cytoreductive surgery (CRS) and hyperthermic intraperitoneal chemotherapy (HIPEC). Angiogenesis is under control of multiple molecules of which HIF1a, SDF1, CXCR4, and VEGF are key players. We investigated these angiogenesis-related markers and their prognostic value in patients with PM arising from CRC treated with CRS and HIPEC.

**Patients and Methods:**

Clinicopathological data and tissue specimens were collected in 2 tertiary referral centers from 52 patients who underwent treatment for isolated PM of CRC. Whole tissue specimens were subsequently analyzed for protein expression of HIF1a, SDF1, CXCR4, and VEGF by immunohistochemistry. Microvessel density (MVD) was analyzed by CD31 immunohistochemistry. The relationship between overall survival (OS) and protein expression as well as other clinicopathological characteristics was analyzed.

**Results:**

Univariate analysis showed that high peritoneal cancer index (PCI), resection with residual disease and high expression of VEGF were negatively correlated with OS after treatment with CRS and HIPEC (*P* < 0.01, *P* < 0.01, and *P* = 0.02, respectively). However, no association was found between the other markers and OS (*P* > 0.05). Multivariate analysis showed an independent association between OS and PCI, resection outcome and VEGF expression (multivariate HR: 6.1, 7.8 and 3.8, respectively, *P* ≤ 0.05).

**Conclusions:**

An independent association was found between high VEGF expression levels and worse OS after CRS and HIPEC. The addition of VEGF expression to the routine clinicopathological workup could help to identify patients at risk for early treatment failure. Furthermore, VEGF may be a potential target for adjuvant treatment in these patients.

**Electronic supplementary material:**

The online version of this article (doi:10.1245/s10434-015-5023-0) contains supplementary material, which is available to authorized users.

Colorectal cancer (CRC) is a major health concern in the Western world. It is the third most common cancer worldwide for both males and females, accounting for more than 1 million new cases and approximately 600,000 deaths annually. In the course of their disease, roughly 25 % of these patients will develop peritoneal metastases (PM), alone or in combination with other metastases.^1^–^3^

In CRC, isolated peritoneal metastases are regarded as a form of localized disease spread and are thus considered amenable to local control, i.e., surgery.^4^,^5^ PM are increasingly treated with curative intent, using cytoreductive surgery (CRS) and hyperthermic intraperitoneal chemotherapy (HIPEC), as opposed to systemic chemotherapy.^6^,^7^ Because treatment with CRS and HIPEC has morbidity and mortality rates of 15–18 % and 5 %, respectively, it is of utmost importance to carefully select those patients who will benefit most from this treatment.^7^–^10^

At present, patients are selected solely based on clinical parameters and intraoperative findings. Based on the hypothesis that phenotype of PM in CRC, and thus also clinical behavior, is driven by underlying biological mechanisms, readouts of disease biology (i.e., biomarkers) will aid in establishing a more refined identification of suitable patients. Additionally, molecular targets may be of great value in prognosis assessment, imaging, and guidance of therapy.

Metastasis formation depends on the combined processes of dissemination of tumor cells and development of a receptive microenvironment. One important condition for successful outgrowth of these tumor cells is the presence of sufficient oxygen, aided by the formation of new blood vessels referred to as angiogenesis.^11^ Angiogenesis is under control of multiple molecules of which HIF1a, SDF1, CXCR4, and VEGF are key players. Vascular endothelial growth factor (VEGF) is the most important and best characterized angiogenic factor and also the target of the anticancer drug bevacuzimab.^12^ The interaction of CXCR4 and SDF1 could advance tumor progression and metastases through the induction of VEGF-mediated angiogenesis.^13^ Furthermore, HIF1 is known to regulate the activation of VEGF directly (See Supplemental Fig. 1).^14^,^15^

Expression of HIF1, CXCR4, SDF1, and VEGF have each been reported to have clinical implications in several malignancies, including primary CRC.^16^ Furthermore, multiple studies have shown the relevance of angiogenesis, measured by the formation of microvessels (i.e., microvessel density [MVD]) in CRC.^17^ Therefore, we hypothesized that these molecules may serve as prognostic markers in this population of metastasized CRC patients.

## Materials and Methods

Patients were included from 2 prospective registries. All consecutive patients treated with curative intent with CRS and HIPEC at the Catharina Hospital in Eindhoven from 2007 to 2010 and from the VU University Medical Center Amsterdam from 2010 and 2011, both tertiary referral centers for patients with peritoneal surface malignancy, were reviewed for inclusion. Only patients presenting with isolated PM were included for this retrospective study.

Clinicopathological data were extracted from the patient records at both institutions. All tumors were staged according to the fifth version of the American Joint Committee on Cancer (AJCC) pathologic-node-metastasis (TNM) classification.

Formalin-fixed and paraffin-embedded (FFPE) tissue specimens obtained during CRS were collected from the archives and hematoxylin-eosin (H&E) slides were reviewed to verify the presence of tumor cells. Collection, storage, and use of clinicopathological data and tissue specimens were performed in compliance with the “Code for Proper Secondary Use of Human Tissue in The Netherlands.”

### Treatment—Cytoreductive Surgery and HIPEC

The preoperative workup and the CRS and HIPEC procedure were carried out in a uniform fashion by both surgical teams according to the Dutch protocol using the open coliseum technique with Mitomycin C.^7^,^18^

### Tissue Specimens and Immunohistochemistry Protocols

The 4-μm sections were mounted on glass slides, deparaffinized, and rehydrated. Endogenous peroxidase was blocked using 0.3 % hydrogen peroxide in methanol. All consecutive slides were subsequently immunohistochemically stained for all markers according to the optimized protocols summarized in Supplementary Table 1. All sections were counterstained with Mayer’s hematoxylin.

**Fig. 1:**
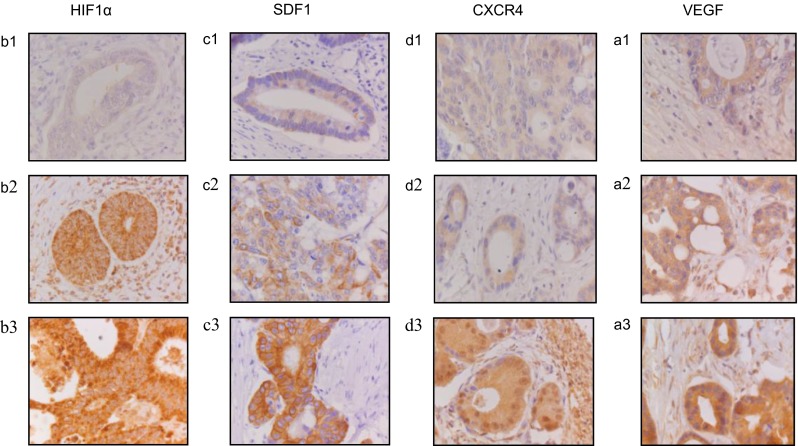
Expression pattern of **a** HIF1a, **b** SDF1, **c** CXCR4, and **d** VEGF staining in peritoneal metastases of colorectal cancer epithelium. Immunohistochemical staining patterns ranged from weak to strong epithelial (nucleus and cytoplasm) staining for all 4 markers. Representative examples of all stainings, ranging from weak (1) to strong (3) in peritoneal metastases epithelium are shown

Scoring was performed using a 10× objective or a 20× objective, depending on whether the staining was cytoplasmic (10× /0.25; diameter 2.01 mm) or nuclear (20× /0.45; diameter 0.98 mm). The intensity observed in the neoplastic cells was subsequently scored as negative, weak, moderate, and strong. For HIF1a, protein expression in the nuclei of tumor cells was scored, whereas for SDF1, CXCR4, and VEGF intensities were scored in the cytoplasm (Fig. 1). All tissue samples were analyzed blinded to corresponding clinicopathological information. A second investigator (GAM) re-evaluated 10 % of the samples in a blinded fashion, and the samples were scored by consensus between the first and second investigators as a quality-control step. Intensity of the staining was subsequently dichotomized, i.e., “low expression” or “high expression” at different cutoffs for 4 markers as shown in Table 1. All analyses were performed using the dichotomized staining intensity score.

**Table 1:** Patient and tumor characteristics

Total number of patients	*N*	%
*Gender*		
Male	23	43.4 %
Female	30	56.6 %
*Age (mean + SD)*	58 years	SD 12.0 years
*Follow-up (median, range*)	22.5 months	0–59 months
*Location primary tumor*		
Colon, including appendix	39	73.6 %
Rectosigmoid	8	15.1 %
Rectum	5	9.4 %
Double tumor	1	1.9 %
*Tumor type*		
Adenocarcinoma	33	62.3 %
Mucinous adenocarcinoma	16	30.2 %
Signet-cell carcinoma	4	7.5 %
*T classification of primary tumor*		
T1	1	1.9 %
T2	1	1.9 %
T3	23	43.4 %
T4	28	52.8 %
*Lymph node status primary tumor*		
Negative	13	24.5 %
Positive	39	73.6 %
Unknown	1	1.9 %
*Timing peritoneal metastases*		
Synchronous	30	56.6 %
Metachronous	23	43.4 %
*Simplified Peritoneal Cancer Index*		
<2	1	1.9 %
2–4	32	60.4 %
5	11	20.8 %
>5	5	9.4 %
Unknown	4	7.5 %
*Resection outcome*		
R0/R1	47	88.7 %
R2	6	11.3 %
*Chemotherapy after CRS and HIPEC*		
Yes	36	67.9 %
No	13	24.5 %
Unknown	4	7.5 %

As for CD31, all specimens were stained using an anti-CD31 antibody according to an optimized protocol summarized in Supplemental Table 1. Finally, the average MVD was quantified in the peritoneal lesions using a computerized morphometric and image analysis approach, as previously described.^19^ In short, complete slides were scanned using a digital Mirax slide Scanner system (3DHISTECH, Budapest, Hungary) with a 20× objective with a numerical aperture of 0.75 and a Sony DFW-X710 Fire Wire 1/3″ type progressive SCAN IT CCD (pixel size 4.65 × 4.65 μm^2^). The scan resolution of all images at 20× was 0.23 μm. After scanning representative areas of the tumor deposits were annotated manually using the Panoramic Viewer software (3D Histech) and subsequently exported in the TIFF image format. A computerized morphometric analysis of the CD31 stained slides was executed, using ImageJ. Subsequently, the MVD was dichotomized as “high” and “low” MVD by setting the threshold at 27 % of the analyzed area stained for MVD, as based on the median.

### Statistical Analysis

Data analysis was performed with the Statistical Package for the Socials Sciences (SPSS Inc., Chicago IL) version 20 for OsX. Descriptive statistics were used to describe clinical and treatment-related factors in the cohort.

The primary endpoint was overall survival (OS), which was defined as time (months) from date of CRS and HIPEC to death from any cause. Survivors were censored on the date they were last known to be alive. For analytical purposes, patients surviving less than 12.6 months post-treatment were additionally categorized as short survivors, and patients surviving more than 12.6 months as long survivors, based on results from the first and only randomized controlled trial comparing CRS and HIPEC and conventional chemotherapy.^7^

Associations between several clinicopathological variables were tested for significance using the unpaired *t* test or the Mann–Whitney *U* test (association between dichotomous and continuous variable, either distributed normally or not normally), and the Chi square test for unpaired ordinal and categorical data. Associations between marker expression and clinicopathological variables were analyzed using the Chi square, Kruskal–Wallis or Mann–Whitney *U* test, depending on the type of variables analyzed. Survival was analyzed using the Kaplan–Meier method. Additionally, established clinicopathological variables were included in a multivariate Cox regression analysis to determine the independent effect of each variable. Input variables were all first tested in a univariate fashion for association with OS, and only significant terms were included in the multivariate model (multivariate Cox regression analysis). The variable selection in the multivariate Cox model was carried out using backward selection with a threshold *p* value for exclusion that was set at 0.1.

A *p* value ≤0.05 was considered statistically significant. All data reported was REMARK compliant.^20^

## Results

The initial study cohort consisted of 53 patients. One patient was lost to follow-up. The patient characteristics are summarized in Table 1.

The median survival for the entire cohort (*n* = 52) was 26 months (Supplemental Fig. 2). A total of 25 events were recorded at the end of follow-up. Univariate analysis showed that tumor burden (simplified Peritoneal Cancer index, sPCI) and resection outcome were negatively correlated with survival after treatment (Supplemental Table 2).^21^

Nine patients were excluded for technical reasons (loss of tissue stained for CXCR4, SDF1, VEGF and CD31 respectively), while for HIF1a ten patients were lost for the same reason. Thus, for final marker analysis 42 patients remained for analysis of HIF1a and 43 patients remained for the analysis of SDF1, CXCR4 and VEGF. For MVD analysis (CD31) data was available from 36 cases (Table 2).

**Table 2:** Low versus high expression of HIF1a, SDF1, CXCR4, VEGF, and MVD

Antigen	Low expression	High expression
HIF1a	Negative, *N* = 0 Weak, *N* = 13 (31.0 %)	Moderate, *N* = 15 (35.7 %) Strong, *N* = 14 (33.3 %)
SDF1	Negative, *N* = 0 Weak, *N* = 3 (7.0 %) Moderate, N = 12 (27.9 %)	Strong, *N* = 28 (65.1 %)
CXCR4	Negative, *N* = 0 Weak, *N* = 2 (4.7 %) Moderate, *N* = 16 (37.2 %)	Strong, *N* = 25 (58.1 %)
VEGF	Negative, *N* = 0 Weak, *N* = 3 (7.0 %) Moderate, *N* = 19 (44.2 %)	Strong, *N* = 21 (48.8 %)
MVD	Low, *N* = 19 (52.8 %)	High, *N* = 17 (47.2 %)

An association was noted between a high HIF1a expression and favorable resection outcome (*p* = 0.03) and male gender and higher CXCR4 expression (p = 0.01) No association was seen between expression levels of HIF1a, CXCR4, SDF1, VEGF, and MVD with the (other) clinicopathological characteristics listed in Table 1 (*p* > 0.05, data not shown). In addition, there was no association between the expression of the 4 markers and the MVD (*p* > 0.05, data not shown).

**Fig. 2:**
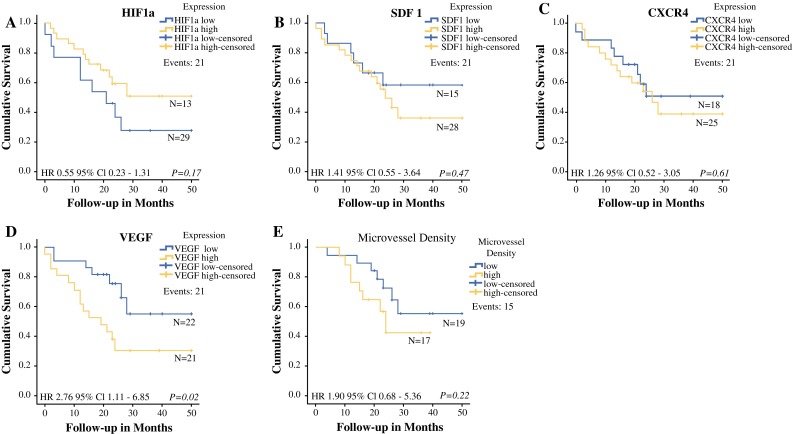
Kaplan-Meier curves showing the correlation between high and low expression of respectively, **a** HIF1a, **b** SDF1, **c** CXCR4, **d** VEGF, and **e** MVD and overall survival in patients undergoing curative CRS and HIPEC for the treatment of PM of CRC

A total of 21 events occurred during follow-up in the group of patients successfully analyzed for protein expression. Only for VEGF a significant difference in overall survival between groups with high versus low expression was observed (mean OS 23.8 months versus 36.1 months, respectively, *p* = 0.02) (Fig. 2). For HIF1a, CXCR4, SDF1, and MVD, there was no significant association between protein expression and OS (*p* > 0.05) (Fig. 2).

In addition, expression for VEGF was associated with short and long survival after treatment with CRS and HIPEC (*p* = 0.02). This was not the case for the other 4 markers HIF1a, SDF1, CXCR4, and MVD (*p* > 0.05).

In the multiple regression analysis, it was found that sPCI, resection outcome and VEGF expression (high versus low expression) were significant independent predictors of survival (*p* = 0.02, *p* = 0.05, *p* = 0.008, respectively). High VEGF expression had a hazard ratio of 3.8 (95 % CI 1.41–10.06), indicating an autonomous association between VEGF expression and OS (Table 3).

**Table 3:** Multivariate analysis of overall survival for the complete CRS and HIPEC cohort (*N* = 52)

Variable	Hazard ratio	95 % CI	*p* value
*Simplified Peritoneal Cancer Index*			0.02
2–4 abdominal regions affected	1.00 (ref)	–	
5 abdominal regions affected	3.01	1.04–8.72	
5–7 abdominal regions affected	6.06	1.28–28.70	
*Resection outcome*			0.05
No residual tumor	1.00 (ref)	–	
Residual tumor <2.5 mm	2.51	0.77–8.20	
Residual tumor >2.5 mm	7.69	1.50–28.70	
*VEGF expression*	3.76	1.41–10.06	<0.01

## Discussion

Isolated peritoneal metastases are increasingly being treated with curative intent by CRS and HIPEC, and with good clinical results.^22^,^23^ However, known clinical factors alone appear to be insufficiently discriminatory for patient selection, as patients are often observed presenting with rapid recurrence after treatment despite having seemingly favorable prognostic clinical profile. The present study revealed an independent association between high VEGF expression levels and worse survival after CRS and HIPEC. The possible addition of VEGF expression to the routine pathological workup could therefore potentially aid in identifying those patients at risk for early treatment failure despite their seemingly favorable clinical profile.

In addition, a correlation was noted between high expression of CXCR4 and male gender. Data in published literature on correlation between gender and CXCR4 expression are scarce and conflicting and thus remain inconclusive.^24^,^25^ Interestingly, we also found an association between high HIF1a expression and a more favorable resection outcome. One explanation, albeit speculative, could be that HIF1a competent tumor cells, i.e., with a relatively high HIF1a expression, behave less aggressively under hypoxic conditions than HIF1 negative tumor cells, because they are still dependent on the blood supply from blood vessels and have not yet (fully) developed the capacity to survive under such circumstances.^26^–^29^ This could also explain the tendency observed toward better OS for patients with high expression of HIF1a (Fig. 2).

Besides the biological connection between these molecules, expression of HIF1a, CXCR4, SDF1, and VEGF have each been reported to have clinical implications in several malignancies. Both lack of HIF1a expression under hypoxic circumstances and overexpression have been previously linked to tumor progression, aggressive biological behavior, and patient prognosis in several types of carcinomas.^26^–^31^ CXCR4 is the most common chemokine expressed in tumors such as ovarian, breast, and colorectal cancer. Its ligand, SDF1, has been described as highly expressed in metastatic sites, such as the lung, lymph nodes, and liver, and has been correlated with grade and prognosis in renal cell and breast carcinoma.^32^,^33^ The prognostic value of high levels of VEGF has been demonstrated in multiple solid tumors and is associated with metastasis in CRC.^34^ In addition, VEGF levels have been reported to predict survival in patients with carcinomatosis arising from several malignancies.^35^–^37^

While in the present study VEGF expression levels appeared to have prognostic relevance, such an association was not observed for HIF1a, SDF1, and CXCR4 in the present cohort. On one hand, this may look counterintuitive, as VEGF is regulated by HIF1a, just like SDF1 and CXCR4, but on the other hand these regulatory networks in vivo are subject to many interactions and apparently in PM, other regulation mechanisms, including oncogenes, of VEGF expression prevail over HIF1a. In fact, the lack of prognostic significance of both SDF1 and CXCR4 in the present study may be consistent with this observation.^16^,^38^

The current finding is also consistent with several other, both preclinical and clinical studies on PM arising from several epithelial malignancies such as ovarian carcinoma in which VEGF has been shown to play a role in PM and prognosis.^35^,^39^–^47^ Several studies have assessed the effect of blocking VEGF both on ascites formation and PM formation and progression.^39^,^41^,^43^,^44^,^46^–^49^ All these studies show that blocking VEGF diminishes both ascites and PM and thus improves survival after surgical treatment. Most of these studies are preclinical, and there are currently no clinical trials specifically addressing the effect of systemic treatment in peritoneal cancer patients. However, a subgroup analysis of clinical trials proving the efficacy of bevacizumab added to standard chemotherapy in the palliative treatment of metastatic CRC suggested that bevacizumab may also be beneficial for peritoneal cancer patients.^50^,^51^ Similar results were retrieved in a population-based study.^51^ These findings have been supported by a recent study, in which 16 % of patients received neoadjuvant treatment including bevacizumab. In this study, the addition of bevacizumab was an independent, favorable prognostic factor for OS after CRS and HIPEC.^52^ These findings, as well as evaluation of possible side effects, await further validation. In a recent study, carried out specifically in the CRS and HIPEC population, one group described the early postoperative major morbidity rate to be significantly higher after the administration of bevacizumab prior to CRS and HIPEC in a cohort consisting of 182 patients, of which 80 received bevacizumab.^53^ However, in a meta-analysis including more than 3000 patients treated with bevacizumab in metastatic CRC the authors concluded the therapy to be effective and the amount and severity of reported adverse effects to be acceptable.^9^

In addition to utilizing VEGF as a treatment target, it can also be used in the improvement of current preoperative and intraoperative imaging. Encouraging results have been reported on the use of specific VEGF tracers (e.g., ^89^Zr-Bevacizumab), which can be used for the visualization of VEGF expression in vivo. These interesting and ground-breaking developments could signal a new era in which the expression of certain molecules, e.g., VEGF, could aid not only in the treatment of our patients, but also in giving the treating CRS and HIPEC surgeon the much needed edge in the operating room by better visualization of even the smallest of tumor deposits.^54^–^56^ Despite limitations of our study such as a limited sample size, we believe the results shown are an important step toward furthering our knowledge of the molecular landscape of PM of CRC. The evidence gathered from (pre-)clinical studies indicate that VEGF expression possibly plays an important role in the pathogenesis of PM. In addition VEGF can be targeted with specific antibodies, and these can also be labeled to improve visualization, both preoperatively and intraoperatively. This increasing evidence supports the notion that this oncogenic pathway deserves further study in this subgroup of metastatic CRC patients.

In conclusion, high expression of VEGF was frequently observed in PM of CRC and in the present cohort higher VEGF expression levels correlated with worse overall survival after curative CRS and HIPEC. This may indicate that VEGF expression may not only serve as a prognostic marker, but also that adding anti-VEGF antibody based therapies, i.e., bevacizumab, could have additional therapeutic value in this subgroup of metastatic colorectal cancer patients.

## Electronic supplementary material

Below is the link to the electronic supplementary material.

Supplementary material 1 (DOCX 769 kb)
