# Supplementary material for: Angiogenesis-Related Markers and Prognosis After Cytoreductive Surgery and Hyperthermic Intraperitoneal Chemotherapy for Metastatic Colorectal Cancer
Source: Ann Surg Oncol. 2016 Jan 4;23:1601–8. doi: 10.1245/s10434-015-5023-0 (PMC4819744; doi:10.1245/s10434-015-5023-0)

**Supplementary Data:**


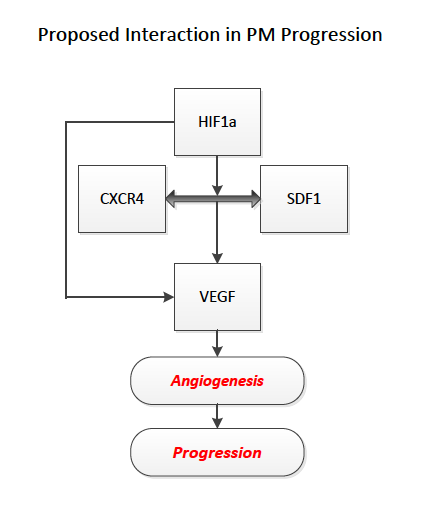


| **Antibody** | **Species** | **Company** | **Dilution** | **Incubation** | **Antigen retrieval** | **Detection** |
| --- | --- | --- | --- | --- | --- | --- |
| HIF1a | Mouse | BD Pharmingen | 1:500 | 30’ RT | WB TRS 97 °C 45’ | CSA |
| CXCR4 | Rabbit | SDIX | 1:1000 | o/n 4°C | MW Citr 15’ | Envision |
| SDF1 | Mouse | R&D Systems | 1:400 | o/n 4°C | MW Citr 15’ | Envision |
| VEGF | Mouse | DAKO | 1:50 | o/n 4°C | MW Citr 15’ | Powervision |
| CD31 | Mouse | DAKO | 1:50 | 60’ RT | MP Tris/EDTA 15’ | Powervision |


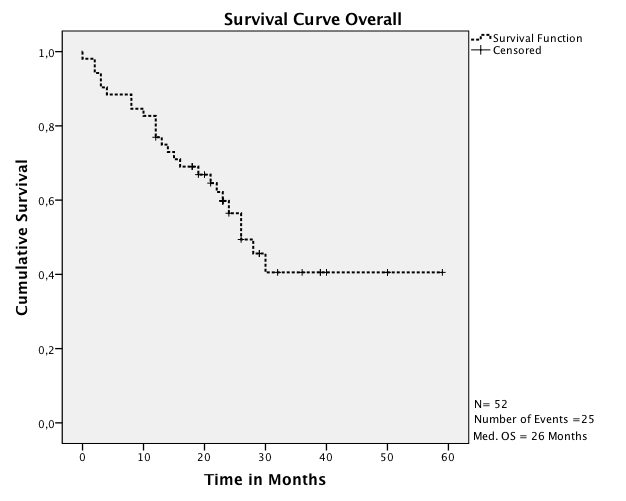


| **Variable** | **P-value** |
| --- | --- |
| *Gender*  *Age*  *Location Prim. Tumor*  *Tumor Type*  *T-classification Prim. Tumor*  *LN status*  *Timing PM*  *Simplified Peritoneal Cancer Index*  *Resection Outcome*  *Chemotherapy after CRS & HIPEC* | NS  NS  NS  NS  NS  NS  NS  ***0.005***  ***0.002***  NS |


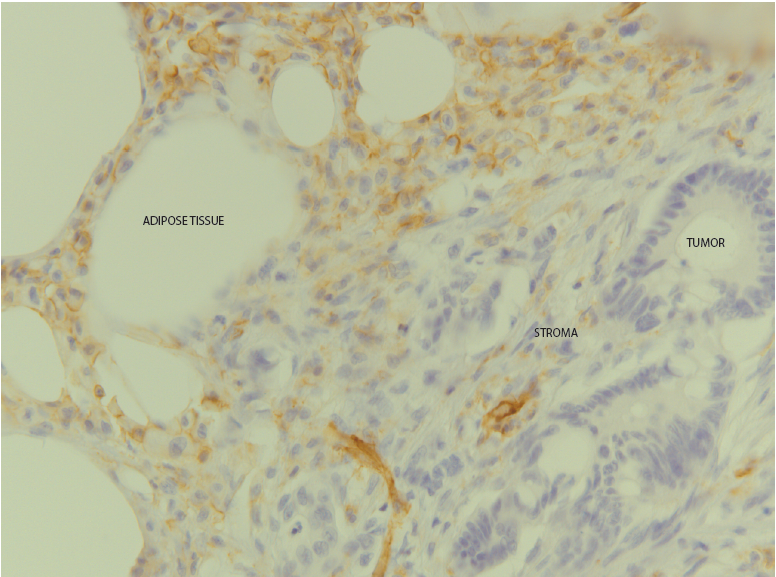

Supplement: Supplementary file 1 — Supplementary material 1 (DOCX 769 kb) [file 10434_2015_5023_MOESM1_ESM.docx]
